# A digital mental health intervention to reduce depressive symptoms among overseas Filipino workers: protocol for a pilot hybrid type 1 effectiveness-implementation randomized controlled trial

**DOI:** 10.1186/s43058-020-00072-y

**Published:** 2020-10-31

**Authors:** Andrian Liem, Melissa R. Garabiles, Karmia A. Pakingan, Wen Chen, Agnes Iok Fong Lam, Sebastian Burchert, Brian J. Hall

**Affiliations:** 1 Department of Communication, University of Macau, Macao, SAR China; 2 Department of Psychology, Ateneo de Manila University, Manila, Philippines; 3 Department of Psychology, De La Salle University, Manila, Philippines; 4 Department of Medical Statistics, School of Public Health, Sun Yat-sen University, Zhongshan Road 2, Guangzhou, China; 5 Sun Yat-sen Centre for Migrant Health Policy, Sun Yat-sen University, Zhongshan Road 2, Guangzhou, China; 6 Department of Communication & Centre for Macau Studies, University of Macau, Macao, SAR China; 7 Division of Clinical-Psychological Intervention, Department of Education and Psychology, Freie Universität Berlin, Berlin, Germany; 8 New York University (Shanghai), Shanghai, People’s Republic of China; 9 Johns Hopkins Bloomberg School of Public Health, Baltimore, USA

**Keywords:** Digital mental health, Telemedicine, Migrant workers, Migrant health, Global mental health, Mixed methods

## Abstract

**Background:**

The current pilot randomized controlled trial (RCT) protocol will comprehensively describe the implementation of a culturally adapted Filipino version of the World Health Organization Step-by-Step (SbS-F) program, unguided online psychological intervention for people with depression based on behavioral activation, among overseas Filipino workers (OFWs) in Macao (Special Administrative Region). The main objective of this pilot study is to explore the preliminary effectiveness of the SbS-F program to decrease participant-reported depressive symptoms compared to enhanced care as usual (ECAU); and the secondary objectives are to explore the preliminary effectiveness of the SbS-F to decrease participant-reported anxiety symptoms and improve wellbeing, and to evaluate the potential for SbS-F implementation in real-world settings.

**Methods:**

This trial will follow an effectiveness-implementation hybrid type 1 trial design and utilize the Reach, Efficacy/Effectiveness, Adoption, Implementation, and Maintenance (RE-AIM) framework to accelerate the translation of clinical research into more effective implementation strategies and policies. Participants will be randomized 1:1 to control and treatment groups. Control group participants will receive ECAU that consists of brief depression psychoeducation and referral to local community partners. Treatment group participants will receive a 5-session of digital intervention through a mobile phone application. The primary outcome (depression) and psychological secondary outcomes (anxiety symptoms and wellbeing) will be measured using validated instruments. To evaluate study implementation, an embedded mixed-methods design will be used to collect data from various stakeholders. Data then will be analyzed using intention to treat principle and reported following the Consolidated Standards of Reporting Trials (CONSORT) guideline.

**Discussion:**

This study will provide important new knowledge about the preliminary effectiveness of SbS-F, a mobile application, as a digital mental health intervention and its scalability. If SbS-F shows positive results among OFWs in Macao, it has strong potential to be used by OFWs in other countries that may also experience depression and difficulty accessing mental health services.

**Trial registration:**

Prospective registration, Chinese Clinical Trial Registry (ChiCTR2000034959) on 26/07/2020.

Contributions to the literature

- The first intervention trial protocol to address mental health problems among international migrant workers during the COVID-19 pandemic.
- The first internet-based intervention trial protocol using a mobile application to improve depressive symptoms among migrant workers (overseas Filipino workers).
- A systematic pilot study protocol to assess the effectiveness, feasibility, and implementation of the Filipino version of the WHO Step-by-Step (SbS-F) program using an implementation science hybrid trial design and Reach, Efficacy/Effectiveness, Adoption, Implementation, and Maintenance (RE-AIM) framework.

## Background

Overseas Filipino workers (OFWs) are among the largest transnational labor migrant populations in the world. There are currently over 2.3 million OFWs working across the globe [1]. Filipinos comprise 15% of labor migrants in the Macao Special Administrative Region (SAR) of the People’s Republic of China [2] where labor migration rose nearly 25% in the last 5 years [3] and mainly worked in low-wage jobs [4].

Previous studies among OFWs point to a high burden of mental disorders. The assumed causes are difficulties and stresses related to migration and labor issues [5]. Exposure to discrimination in particular was linked to higher depression among Filipino migrants [6]. This was also reported in a study conducted in Macao, which showed that common mental disorders are likely to contribute to a significant burden to this population [7].

In addition, the prevalence of depression and anxiety were over 30%, and for posttraumatic stress disorder (PTSD), it was over 25% based on initial studies to validate screening tools and establish initial prevalence estimates in advance of an epidemiological study [8–11]. These prevalence estimates were more than two times higher than in the local Macao Chinese population, providing evidence of mental health inequalities [12]. The burden of mental disorders for OFWs living in the Macao SAR is of significant concern, especially since there are no known treatment options currently available to address the mental health of this community in this context. Furthermore, migrant workers’ mental health may worsen during the COVID-19 pandemic and their health needs may not be adequately addressed by the host country [13].

Digital mental health interventions are promising intervention programs to address the mental health burden in contexts where there are few mental health providers [14]. For example, a randomized controlled trial in Indonesia demonstrated that a guided self-help intervention reduced depression symptoms [15]. Guided self-help internet-based interventions are shown to reduce depressive symptoms [16], including smartphone-based interventions that showed small to medium effect size [17]. These programs ideally serve the needs of the majority of the population who may have mild to moderate depressive symptoms. As public mental health interventions, unguided self-help programs are likely to reach the largest number of people who need support, but for whom interventions are either not available or desirable.

At present, there is no known digital mental health intervention program designed or implemented for use among OFWs. One previous study among OFWs in predeparture trainings in the Philippines reported that a high proportion would intend to use an online counselling program if it were made available [18]. A subsequent population study among OFWs in Macao found that over 90% owned a smartphone and more than 60% of participants would utilize the digital mental health intervention if it were made available [19]. This suggests that for Filipino OFWs who may need assistance with mental health, a digital mental health solution through a mobile phone application may be useful, especially in light of the current pandemic situation.

## Current study

### The World Health Organization (WHO) Step-by-Step (SbS) program

The WHO developed Step-by-Step (SbS), a minimally guided online technology supported-intervention for people with depression based on behavioral activation [20]. The SbS program is an integral part of the WHO expansion into developing and testing scalable psychological interventions in contexts and for populations with limited access to needed mental health services. The prototype of Step-by-Step was developed from Problem Management Plus (PM+), a lay health worker delivered WHO intervention that has been trialed among people affected by terrorism and war in Pakistan [21] and Kenya [22]. The program was shown to be effective in reducing anxiety, depression, and posttraumatic stress among people with PTSD in Pakistan.

The SbS program with minimal guidance has been piloted among Syrian refugees and local population in Lebanon [23]. A qualitative evaluation of the SbS program in Germany found that the Syrian refugees accepted the digital intervention and perceived the potential impact of the intervention on their mental health [24]. As part of the EU-funded STRENGTHS project [25], Step-by-Step is currently being evaluated as a self-guided intervention with a contact-on-demand guidance model. The current study in Macao uses a similar guidance model to the one used in the STRENGTHS trials and adapted the intervention content for OFWs with a rigorous cultural adaptation method [26].

The approach in this cultural adaptation process involved consultations with Filipino psychologists who are experts in providing treatment to OFWs. Following these consultations, the program was culturally adapted through intensive weekly focus group meetings with male and female OFWs from a variety of occupational backgrounds. After adapting the text and illustrations, the changes were shown to the OFWs who again provided feedback and approval. The entire program was then translated into the Filipino language and was once again shown to OFWs for their suggestions and comments. The results of this exercise were shared with community stakeholders (e.g., non-government organization [NGO] staff, mental health practitioners, Philippine Consulate staff) for their final comments. The final product is a fully adapted Filipino version of the WHO SbS (SbS-F) scalable intervention program.

A small uncontrolled feasibility study was conducted to evaluate recruitment and screening methods and evaluate the guidance model and trends in symptom change [26]. Initial results demonstrated that social media-based recruitment is most likely to succeed, supplemented with word of mouth through community influencers and direct recruitment methods through referrals from local NGOs, the Philippines Consulate, and pastoral counsellors. Our screening process was successful and there was sufficient interest and demand for the program to warrant a full-scale randomized controlled trial. Minimal guidance of the intervention was tolerable to participants. Therefore, the SbS-F program has the potential to address psychological distress experienced by OFWs in Macao. Furthermore, this innovative digital mental health intervention provides an innovation to lessen the treatment gap due to very limited mental health services in Macao [12].

### Hybrid effectiveness-implementation design

**Fig. 1:**
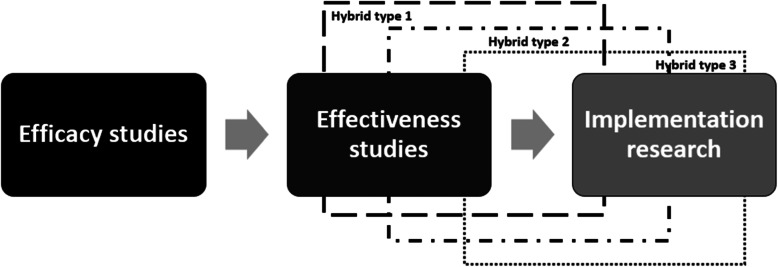
Hybrid effectiveness-implementation designs

The traditional approach for research translation from efficacy and effectiveness trials to implement the findings into practice has been criticized because it is time consuming and provides less-effective implementation strategies [27, 28]. An innovative hybrid effectiveness-implementation design has been introduced to accelerate the translation of clinical research into more effective implementation strategies and policies [29]. The novelty of this design is a dual focus a priori in evaluating clinical effectiveness and implementation. The hybrid effectiveness-implementation design has three types, which differ on a continuum with a focus varying between effectiveness and implementation [30]: type 1 is more focused on effectiveness than on implementation strategy, type 2 balances its focus between effectiveness and implementation, and type 3 is more focused on implementation strategy then effectiveness outcomes. An illustration of these types can be found in Fig. 1. However, to discuss in detail the range of hybrid effectiveness-implementation designs is beyond the scope of this protocol and it has been reported elsewhere [27–29].

This current pilot study will use a hybrid effectiveness-implementation type 1 design because the larger attention will be given into the SbS-F program effectiveness in improving mental health of OFWs in Macao, followed by a minimal evaluation of its implementation within a non-profit organization’s practice. In order to utilize this trial design, the intervention should be supported by at least indirect effectiveness evidence and pose minimal risk to participants [29]. These characteristics are found in the WHO SbS program, since it was trialed on Syrian refugees, who share similar background as immigrants with OFWs in Macao, and showed no perceived risks for its application [23], and the evidence from the cultural adaptation and initial feasibility study conducted among OFWs in Macao.

To support the hybrid design, the Reach, Efficacy/Effectiveness, Adoption, Implementation, and Maintenance (RE-AIM) framework will be utilized because it is a well-recognized tool that improves the sustainability and scale-up of evidence-based practices [30, 31]. The first domain is Reach to evaluate participation and representativeness of the trial. Second, Efficacy/Effectiveness measures the impact of the program on outcomes. The third domain is Adoption to understand whether the program can be adopted with ease and minimal modification. Fourth, Implementation will uncover whether the intervention has been implemented as planned, including issues and barriers to implementation. Lastly, Maintenance will address the sustainability of the intervention over time. Details of how the RE-AIM framework will be addressed in this trial are found in the “Material and methods” section.

### Study objectives and research questions

The main objective of this pilot study is to explore the preliminary effectiveness of the Filipino version of the WHO Step-by-Step (SbS-F) program to decrease participant-reported depressive symptoms compared to enhanced care as usual (ECAU), and the secondary objectives are to explore the preliminary effectiveness of the SbS-F to decrease participant-reported anxiety symptoms and improve wellbeing, and evaluate the SbS-F’s potential for implementation in real-world settings. Five research questions addressed in this study are:

1. Compared with ECAU, does the SbS-F program demonstrate preliminary evidence of a greater reduction in participant-reported depression symptoms?
2. Compared with ECAU, does the SbS-F program demonstrate preliminary evidence of a greater reduction in participant-reported anxiety symptoms and improved wellbeing?
3. Can the SbS-F program be feasibly adopted into the local community partner’s practice to allow scale-up?
4. Can the SbS-F program be implemented as intended during the study?
5. Can the SbS-F program implementation be maintained after the study concludes?

## Material and methods

### Overview of study design

**Fig. 2:**
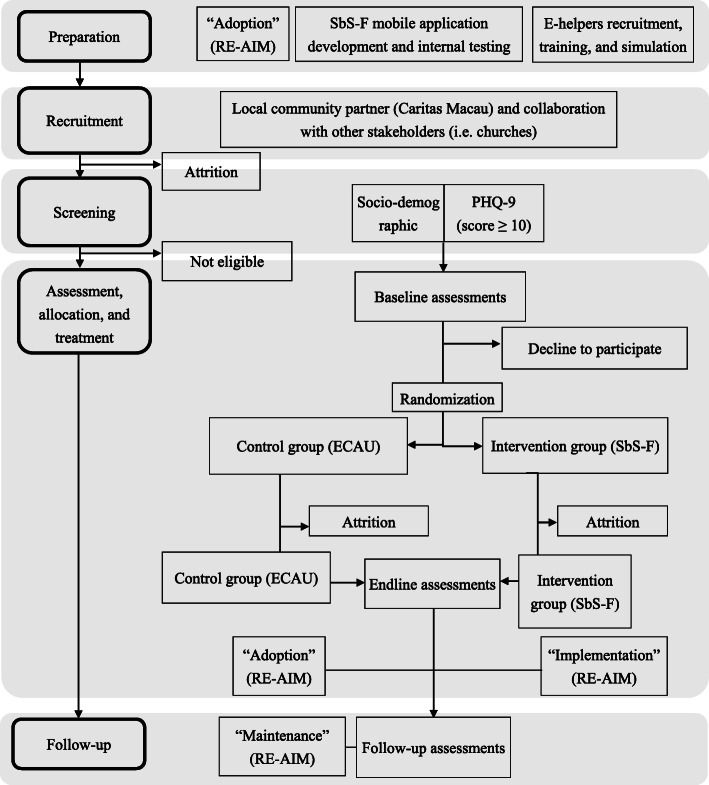
Overview of SbS-F hybrid trial study design following CONSORT diagram

The pilot study will follow the structure of an effectiveness-implementation hybrid type 1 trial [29] with an embedded mixed-methods design [32] as presented in Fig. 2. We adopt the Consolidated Standards of Reporting Trials (CONSORT) guideline. As a part of implementation evaluation, qualitative methods will be embedded into the quantitative experimental design, including interviews and focus groups with participants as well as key local stakeholders. User experiences with the intervention will be explored in the interviews, including elements that were liked or not liked, any issues around treatment acceptability, and possible enhancements to consider for further adaptation. The Reach, Efficacy/Effectiveness, Adoption, Implementation, and Maintenance (RE-AIM) framework from previous hybrid trials [31, 33, 34] will be adopted in this mixed-methods study to successfully achieve the objective of effectiveness-implementation trial as displayed in Table 1.

**Table 1:** The Reach, Efficacy/Effectiveness, Adoption, Implementation, and Maintenance (RE-AIM) framework

Domain and outcome (example)	Source
**R**each • Participant’s and decliner’s characteristics (e.g., compare sociodemographic information between participant and nonparticipant OFWs; reasons for not participating; the most effective recruitment strategy)	• Administrative data • Open-ended questions in a questionnaire
**E**ffectiveness • Primary outcome (depression severity) • Secondary outcome (anxiety and wellbeing) • Reason for withdrawing	• Questionnaires • Interviews and focus groups
**A**doption • Ineligibility reasons • Barriers and enablers of SbS-F adoption into community partner’s program and recommendations for addressing barriers and maximizing adoption • Community partner’s knowledge and beliefs about the SbS-F program	• Administrative data • Questionnaires • Interviews and focus groups
**I**mplementation • Participants’ adherence to the intervention (e.g., duration in accessing the SbS-F and reasons for missing sessions) • Participants’ perceived benefit from the intervention • Barriers and enablers in implementing the program and modifications needed to maximize the implementation (from participants’ and staff’s perspective) • Relevance of the SbS-F story and techniques in participant’s lives • Time and resources required for supporting the program (e.g., number of e-helpers needed and their total working time; e-helpers’ training, selection, and retention)	• Administrative data • Questionnaires • Interviews and focus groups
**M**aintenance • Attrition rates • The durability of intervention effects on depression • Resources, strategy, and policy needed to integrate and maximize the SbS-F intervention into community partner’s program after the study ended	• Administrative data • Questionnaires • Interviews and focus groups

### Setting and timeline

This pilot trial will take place in Macao among OFWs over 12 months (Appendix 1). The total population of OFWs in Macao as of March 2020 is 34,034 (women 65.3%) [35]. Most of OFWs (50.5%) are working in domestic work, followed by hospitality (18.1%), real estate, renting, and business activities (14.9%), and recreation and gaming (6.9%) industries [36]. A population survey among female Filipino domestic workers in Macao [19] found that their median income was MOP 3700 (∼ USD 460; (*IQR* = 3500–4000) per month and the median time working as an OFW was 6 years (*IQR* = 2–11). The median of working time was 67.8 h per week (*IQR* = 52–78) with 4 days off per month. Most participants owned a smartphone (91.3%) and could access internet from their smartphone (77.9%). Additionally, more than 75% of participants can access Wi-Fi to reduce the use of their own data plan. Only 12.6% of participants reported no intention to access professional mental health services.

However, to the authors’ knowledge, there is no official record on migrant workers’ use of health services, including mental health services, and a lack of clear information regarding migrant workers’ health insurance and its coverage for mental health services. In addition, progress in available mental health services of Chinese mental health care system is still only found on government structural level and applied in few big cities in the mainland China for its citizens [37]. As a result, migrant workers (including OFWs) in Macao may face challenges to treat mental disorders that arise during their work period. For example, there is only one public hospital available and the psychology/psychiatry department is only open from Monday to Friday, during times that migrant workers usually work. Alternatively, migrant workers can access a private hospital for mental health services in the weekend. But the fee in the private hospital is 10 times higher than public hospital and this fee roughly equals with 1/6 of their monthly income for one session of psychotherapy.

### Local community partner

This pilot trial will be conducted in collaboration with a local community partner, Caritas Macau (CM). CM was founded in 1951, originally named Centro Social Mateus Ricci, by a Jesuit priest to serve refugees in Macao by providing food supply, job hunting, and document processing [38]. When the refugees had migrated to other countries in the 1960s, CM shifted its services for the poor and homeless elderly. In 1971, CM formally became a member of Caritas international and expanded their services, including training for social workers. Today, CM is offering various programs and assistances for all communities, including migrant workers, in Macao. Some of these CM services are related with mental health and wellbeing. Additionally, CM has established a strong connection with OFWs in Macao through their services and outreach community program (e.g., mental health workshop and relationship counselling), in which participants are predominantly (up to 90%) female OFWs. More detail about local community partner’s service related to mental health will be presented in “Intervention and control groups” section as enhanced care as usual (ECAU) condition.

### Intervention

#### Intervention and control groups

The digital mental health intervention in this study is a mobile and web application named Step-by-Step for Filipino users (SbS-F). The original SbS program was developed by experts in the field of psychology, psychiatry, and global mental health in concert with colleagues at the WHO [20]. Behavioral activation (BA) is the primary treatment component in the intervention, which is simple and effective at reducing depressive symptoms [39, 40] and can be delivered digitally with minimum assistance [41]. Previous trials (e.g., [15, 42]) demonstrated that BA could be delivered by lay health workers, in person and digitally, to reduce anxiety, depression, and PTSD. Also, BA for PTSD among American veterans was associated with greater reduction in depressive symptoms and higher treatment satisfaction than treatment as usual [43].

The SbS-F program content is delivered through a series of illustrated sessions that teach users specific skills that can be applied to reduce psychological distress (Appendix 2). The sessions articulate into an overall story which is delivered by two main characters, available in female and male versions. They guide the user through the intervention content in an interactive, supportive, and culturally appropriate manner. As informed by our cultural adaptation, the first main character is a wise community elder and the second character is a younger person who applied the Step-by-Step techniques themselves.

Each character teaches the user about the program, the intervention techniques and why they work, and model how to apply them through easy to follow real-world examples. The community elder character also teaches the user techniques in greater detail and coaches on how to complete intervention activities. This type of illustrated and adapted format is shown to be an effective digital mental health intervention format [44]. The five therapeutic sessions are summarized in Table 2 below. The intervention will be available online (in mobile application format) with “contact-on-demand” support from trained research assistants called e-helpers. For participants in the intervention group, e-helpers will contact them to explain briefly the SbS-F application.

**Table 2:** Key intervention information

Week	Control group	Intervention group
1	Baseline assessment	Baseline assessment
2	Brief psychoeducation and referral	Onboarding (introduction to the SbS-F mobile application)
3		Session 1: Get started (psychoeducation and trying small and pleasant activities)
4		Session 2: Get active (behavioral activation)
5		Session 3: Beat obstacles (more complex behavioral activation and relaxation)
6		Session 4: Get together (increasing social support)
7		Session 5: Keep it up (relapse prevention).
8	Endline assessment	Endline assessment
20	Follow-up assessment	Follow-up assessment

*Note*. *SbS-F* Step-by-Step program (Filipino version)

On the other side, participants in control group will receive ECAU, which consists of brief psychoeducation on depression and a referral to CM services. This basic depression psychoeducation is formulated based on the information provided during the first session of SbS to assure that the information is same between two groups. A previous study found that online psychoeducation reduced depressive symptoms among adults in Indonesia [15]. In addition, participants will receive information on services provided by CM. There are two CM departments named WelAnser Centre (WC) and Life Hope Hotline (HH) that can be accessed by participants. In WC, there are three social workers with Bachelor of Social Work degree who handle mental health problems of migrant workers with supportive counselling services. These social workers received mental health-related education in their bachelor’s degree and practical mental health care trainings through the Macao Social Welfare Bureau. If the issue is beyond the social worker’s expertise or CM’s scope of service, the client will be referred to another agency. For example, a client who needs medication will be referred to the hospital and accompanied by the social worker as needed.

Due to geographical and time difficulties, not all OFWs can visit the WC to have counselling. Alternatively, they can contact HH for emotional support through phone counselling that is available 24/7. The phone counselling provided by HH is available in Cantonese, Mandarin, and English, with counsellors from a social work or psychology bachelor’s degree background. Moreover, every counsellor at HH has been trained about mental health and crisis intervention. In addition, there is a regular supervisory meeting to discuss any challenges faced by the counsellors. Similar with the counsellors at WC, if the client’s problem is beyond their expertise or CM’s available services, the counsellor will provide client information about other agencies that may be able to assist their needs.

#### E-helpers

The e-helpers will assist in the use of SbS-F mobile phone application and remind participants to log in if they show inactivity. The e-helpers will also identify cases requiring a higher level of care than what SbS-F is designed to deliver. When a high-risk case is identified, the e-helper will encourage the user to contact local supportive services and inform the clinical supervisor. The results of the SbS-F website version feasibility study suggested that a minimal guidance model that included asynchronous on-demand support was sufficient to maintain adherence and retention.

Filipino e-helpers will provide technical and motivational support to OFW users. These e-helpers could be community health workers, graduate students, or upper-level undergraduate students under the supervision of their professor. The e-helpers’ training and guidance will be conducted as a part of a student practicum experience, so students will receive course credit for their participation (Appendix 3). This will be coordinated and supervised by our academic partners at De La Salle University in the Philippines.

E-helper support consists of guiding users through the intervention and supporting them in conducting their own activities. This on-demand guidance will be conducted by the email-like message system (a delay in response) available within the mobile application. This support will be available to users upon their request and users will be allocated to a particular slot and e-helper by the supervisor based on availability and preference. The e-helpers also need to be able to attend weekly supervision sessions with a clinical supervisor. A clinical supervisor will supervise e-helpers and, in emergency situations, provide support, ensure the guidance model was being followed, and troubleshoot any difficulties. Supervision ideally would take place during weekly group meetings, where cases are presented and issues are discussed.

The e-helpers will need to be available for training. Training on digital mental health and scalable psychological interventions will be provided for all e-helpers. This will include a broader orientation to global mental health and the WHO scope of work to address the burden of mental ill health through scalable intervention models. The e-helpers would be also trained on the intervention and how to facilitate/guide the intervention remotely using asynchronous message system and audio call support.

### Sample size

Based on previous behavioral-activation smartphone application RCT study [41], the expected pragmatic total sample size for this pilot study is 53 participants for each group. This sample size target is necessary for a pilot hybrid type study to evaluate the potential challenges for SbS-F implementation in the real-world settings [45]. Estimating the effect size is not an aim of this pilot study because preliminary effectiveness of SbS-F asked in research questions 1 and 2 are exploratory. To achieve this total sample size of 106 participants, 650 participants are targeted for recruitment in anticipation of attrition that is summarized in Fig. 2 of CONSORT flow diagram. From a previous population study with OFWs in Macao [11, 19, 46], it is predicted that 40% of screened participants will be eligible for baseline and 50% of participants during the intervention stage will drop out.

### Recruitment

Participants for SbS-F intervention will be recruited through assistance of a local community partner (CM), for example, on the Caritas Open Day event. Members of our research group will attend the event, together with staff of CM, to explain the intervention program and invite OFWs to participate in the screening process. Together with CM, recruitment will also be conducted at churches by the assistance of the priests who will inform parishioners about the study. Additionally, the research assistants will distribute flyers that contain a link and QR code to the screening survey after the weekly mass. Churches are chosen because the majority of Filipino are Christian/Catholic [47]. For the qualitative methods, purposive sampling technique will be used to select participants with particular characteristics (e.g., by sex and by participants’ involvement during the intervention). The participant numbers of qualitative methods will be determined following the number of participants in trial and established methods for qualitative research [48]. Online recruitment via social media is also an effective strategy used during the feasibility study [26, 49]. Facebook recruitment advertisements and other targeted social media messaging via opinion leaders within the Filipino community will also be used.

### Randomization and blinding

Participants will be randomized by the built-in algorithm in the mobile app with 1:1 ratio to the treatment (SbS-F) and control (ECAU) arms. The algorithm is designed for permuted block randomization where the total number of participants is set prior to the randomization. The algorithm will also randomly choose the length of the block (2, 4, or 6) and the order within a block is also randomly assigned. For illustration, 20 participants can be randomized into this scenario where “T” is for treatment and “C” is for “Control” group: [TCCT], [TTCTCC], [CT], [TC], [CTCT], and [CT]. As a part of the consent process in the screening stage, participants will be informed that this is an intervention study where they might be assigned to different treatment groups. Due to the characteristics and design of this study, blinding of treatment assignment and outcome assessments will not be feasible.

### Participant eligibility

Male or female OFWs will be included if they meet the following criteria: (1) aged 18 or over; (2) understand English and/or Tagalog; (3) holds a valid working visa in Macao; (4) will stay in Macao for at least 6 months more; (5) have a mobile phone; (6) have internet access; and (7) score ≥ 10 on the Patient Health Questionnaire (PHQ-9). OFWs will be excluded from the study if they (1) have suicidal intent based on an item of suicidality screening (“In the past month have you had serious thoughts or a plan to end your life?”); (2) are not physically present in Macao during the study; and (3) are younger than 18 years old.

### Assessments

There will be five measurement time points: (1) screening, (2) baseline, (3) monitoring, (4) endline, and (5) follow-up. A summary of assessment measurements in this study is presented in Table 3 below following standard protocol items for clinical trials (SPIRIT, Additional file 1). Participants in both trial arms will receive compensation for their time and costs associated with required internet use at screening, endline, and follow-up (roughly 6 USD for screening, and 12 USD for endline and follow-up).

**Table 3:** Outcomes and time points of measurement following the SPIRIT guidelines

Outcome	Instrument	Screening	Baseline	Monitoring	Endline	Follow-up
Week			1	3-7	8	20
Sociodemographic (age, education level, sex, marital status, occupation, years as OFW, years of worked in Macao, salary, visa status)	Demographic questions	x	x			
Source of getting SbS-F information	Demographic questions	x				
Mental health care service history	Demographic questions	x	x	x	x	x
Intention to stay in Macao	Demographic questions	x	x			
Smartphone ownership	Demographic questions	x	x			
Internet access	Demographic questions	x	x			
Contact details	Demographic questions	x				
Convenient contact times	Demographic questions	x				
Depression symptoms	PHQ-9	x	x	x	x	x
Anxiety symptoms	GAD-7		x		x	x
Wellbeing	WHO-5		x		x	x
“Adoption” (RE-AIM)	Questionnaires Interviews and focus groups	x			x	
“Implementation” (RE-AIM)	Questionnaires Interviews and focus groups				x	
“Maintenance” (RE-AIM)	Questionnaires Interviews and focus groups					x

*Note*. *GAD-7* Generalized Anxiety Disorder-7, *OFW* overseas Filipino worker, *PHQ-9* Patient Health Questionnaire, *RE-AIM* Reach, Efficacy/Effectiveness, Adoption, Implementation, and Maintenance framework, *SbS-F* Step-by-Step program (Filipino version), *WHO-5* WHO-5 Wellbeing Index

#### Demographic information

In the screening, the participants’ sociodemographic and personal information will be administered first by combining open and closed questions. Samples of open questions are participants’ age, years as OFW, and salary. For closed questions, participants will be given options to choose from, for example, education level (elementary / high school / technical school / some college / college degree or higher), intention to stay in Macao at least 6 months more (yes / no), and access to internet on a smartphone (yes / no). In the Baseline session, participants will be asked again about their sociodemographic and personal information to confirm their eligibility.

#### Primary outcome measurement

The primary outcome is self-reported depression symptoms that will be measured with Patient Health Questionnaire-9 (PHQ-9), a self-administered, relatively short and simple depression scale drawn from the full Patient Health Questionnaire [50]. It is designed to measure depressive symptoms according to the DSM-IV criteria [51] and DSM-5 since no significant difference between DSM-IV and DSM-5 for depression diagnosis criteria [52]. Items are rated from 0 (not at all) to 3 (nearly every day) and scores on each item are summed such that the total score ranges from 0 to 27. Higher scores represent greater depressive symptom severity. The Filipino version of PHQ-9 has been validated for use among female migrant domestic workers [9, 11] and showed high reliability (*α* = .93).

#### Secondary outcomes measurements

Two secondary outcomes of intervention preliminary effectiveness are self-reported anxiety symptoms and wellbeing of participants. The anxiety symptoms will be measured with the Generalized Anxiety Disorder-7 (GAD-7), which was originally developed among primary care patients [53]. The GAD-7 has been translated into various languages and adapted to different contexts, has good psychometric properties, and has good sensitivity and specificity to detect anxiety depression [54]. Items are rated from 0 (not at all) to 3 (nearly every day), and scores on each item are summed such that the total score ranges from 0 to 21. Higher scores represent greater anxiety symptom severity. The Filipino version of GAD-7, which will be used in this study, showed good internal consistency (*α* = .80) in studies among Filipino domestic workers [11, 55].

The psychological wellbeing of participants will be measured with the WHO-5 Wellbeing Index (WHO-5) that has non-invasive questions with high sensitivity in capturing positive affect [56, 57]. Items are rated from 0 (none of the time) to 5 (all of the time), and scores on each item are summed; the total score ranges from 0 to 25. Higher scores represent greater sense of wellbeing. A cross-sectional study in Taiwan that used the WHO-5 found that Filipino migrant care workers reported higher psychological wellbeing than caregivers from Vietnam and Indonesia [58]. The WHO-5 has been also used among Filipino older adults and showed good internal consistency (*α* = .88) [59].

#### Implementation measurements

As a hybrid type 1 trial, this study will assess the SbS-F potential implementation by following the RE-AIM framework as presented in Table 1 with a mixed-methods approach. The quantitative data will be sourced from the administrative information (i.e., duration in accessing the SbS-F application) and questionnaires adopted from a previous hybrid trial of interpersonal psychotherapy for prisoners with depression [45].

The Stakeholder Acceptability Survey (SAS) [60] will be used for assessing ease of delivering SbS-F, the perceived helpfulness of SbS-F, level of enthusiasm for SbS-F, and issues related to sustainability. The stakeholders’ attitudes toward evidence-based practice will be assessed with the revised Evidence-Based Practice Attitude Scale (EBPAS-50) [61]. The stakeholders’ attitudes, knowledge, and skills needed to provide high-quality mental health care will be assessed using Competency Assessment Inventory (CAI) [62]. The perceived barriers and facilitators of implementation from the stakeholders’ perspective will be measured with the Dimensions of Organization Readiness—Revised (DOOR-R) survey [63].

The qualitative data will be gathered through semi-structured individual interviews and focus group discussions with the participants and stakeholders (e.g., staff of local community partner and e-helpers). The interviews and focus group discussion guidelines draft can be found in Appendix 4 and will be finalized following completion of SbS-F intervention since this is an iterative process of qualitative enquiry.

### Ethical considerations

#### Ethical and safety considerations

The SbS-F in this study is a non-pharmacological digital mental health intervention. Similar interventions have been found to be safe for treating depression [64, 65]. The protocol of this hybrid trial has been approved by the Research Ethics Committee at the University Macau (SSHRE19-APP074-FSS) on 16/01/2020 and has also be registered publicly (Chinese Clinical Trial Registry, ChiCTR2000034959) on 26/07/2020.

This treatment is unlikely to induce adverse effects but the severity of depression will be monitored regularly using PHQ-9 following self-harm protection protocol for a study among adults with depression [34]. If a participant develops into a high-risk case (i.e., scoring 2 or above on item 9 of the PHQ-9) during the intervention period and endline, an e-helper will refer the participant to local counselling services and be followed-up. Additionally, participants who answer 2 or above on item 9 of the PHQ-9 will receive a pop-up message on their screen explaining that they may need additional mental health support. Participants who are excluded in the screening because of suicidal ideation will also be referred to local counselling services. The e-helpers will also refer the participants deemed as moderate- and high-risk cases to counselling services and report to the e-helper supervisor and research staff.

Participants who show inactivity in several days (i.e., 7 days) will be sent a message and up to three audio call follow-ups. Participants who indicate that they wish to discontinue the intervention will be invited to provide their e-helper with a reason for discontinuation. The answers from this process will be used in implementation evaluation, particularly on barriers for participation. If participants state that the discontinuation is due to intensified distress, appropriate alternative referrals will be discussed with the participants and initiated when necessary.

The intervention also seeks to ensure no participants will experience stigma from participating. The content of the intervention will look like illustrated story within the mobile application and any sensitive or potentially culturally offensive wordings are removed or reworded based on our completed qualitative cultural adaptation [26].

There is no identified risk in the qualitative interviews with participants and stakeholders. However, interviewers will be trained in responding to distress as a result of interview questions. Should distress arise, interviewers will provide participants a list of contacts for counselling services for additional support.

#### Data management

All data in the study will be collected electronically and will be held securely on password-protected computers that can be accessed only by the members of the research group. To protect participant confidentiality, unique anonymous study IDs will be used for data storing, tracking, and reporting. The data cleaning process will actively search for errors in a planned way. Multiple imputation will be used to handle missing data as its validity in handling missing data in RCT and is available for most types of data [66].

### Data analyses

#### Quantitative data

The intention to treat principle will be used to analyze the intervention preliminary effectiveness with primary and secondary outcomes, which include all participants randomized to a treatment arm regardless of treatment adherence, attrition, or the completion of outcome assessments. The preliminary analysis will be conducted to examine the distribution of variables for skewness, variability, missing data, and outliers, following previous internet-based intervention on depression [15]. Participants’ sociodemographic information and depression severity will be compared between groups using statistical analyses depending on the variable.

The descriptive statistical analyses (i.e., central tendency and dispersion tendency) and graphical methods will be used to summarize baseline characteristics of all key variables. For example, mean, standard deviation, and range of the total number of participant’s log-in activities will be calculated to represent participants’ engagement in the SbS-F program. The quantitative data from implementation measurements (i.e., stakeholders’ attitude toward evidence-based practice) will also be analyzed with descriptive statistical analyses. Comparison of primary and secondary outcomes between groups (control vs intervention) from baseline to follow-up measurements will be performed using a general linear mixed model with adjustment for possible confounders (i.e., and time effect). In particular, the following sociodemographic variables will be controlled as previous studies [7, 59] showed its association with depressive and anxiety symptoms among Filipino communities: sex, marital status, educational level, income level, and language proficiency. The difference of outcomes between the two groups will be covered within the 95% confidence interval for the effect size. The effect sizes (Cohen’s *d*) will be calculated by dividing the estimated mean difference (from baseline, monitoring, endline, to follow-up) reported from the linear mixed model results by the pooled standard deviation (*SD*) at baseline. The effect size will be calculated to inform the power calculations for the planned definitive trial.

The size and pattern of missing data and the reasons will also be documented. Missing data will be analyzed that will investigate the associations between (1) observed variables and patterns of missingness, (2) treatment conditions on rates of missingness and time to missingness; and (3) baseline characteristics and missingness [45]. The sensitivity analyses of treatment completers (participants who finished at least three of five SbS-F modules) will also be measured with the general linear mixed models. The relative risk (RR) and 95% confidence intervals (CIs) for the number of patients who recover from depressive disorder in the SbS-F compared with the ECAU group will be reported in the sensitivity analyses. All the quantitative analyses were performed using SAS Statistical Package 9.4 (SAS Institute Inc., Cary, NC).

#### Qualitative data

All individual interviews and focus group discussions records will be transcribed. The qualitative data then will be analyzed with deductive thematic analysis framework based on the RE-AIM domains assessed, which will be used to develop an initial codebook. The data will be coded separately by stakeholder group (e.g., participants, e-helpers, and community partner staff). An iterative approach will be used in analyzing the qualitative data where two coders will double-code all transcripts and discuss the discrepancies until consensus is reached. A master codebook will be entered into a qualitative analysis software.

## Discussion

Over 30% of OFWs in Macao reported depression and anxiety and more than 25% for PTSD [8–11], which are more than two times higher than in the local Macao Chinese population [12]. However, mental health services in Macao for OFWs are very limited and more expensive compared with services for local people. A digital mental health intervention may be useful in addressing OFWs’ psychological distress. Therefore, this study aims to evaluate the effectiveness of the SbS-F mobile application intervention, compared to ECAU, and to evaluate the SbS-F’s potential for implementation in real-world settings through collaboration with local community partner.

To our knowledge, this is the first intervention trial of online mental health intervention ever conducted in Macao, or among OFWs. The hybrid type 1 effectiveness-implementation randomized controlled trial is chosen to accelerate the translation of clinical research into more effective implementation strategies and policies, especially within the rapid development of technology and information system [29]. Specifically, RE-AIM framework and embedded mixed methods are used to support this hybrid effectiveness-implementation trial in decreasing the discrepancies from trial findings to sustainability of evidence-based practices [30–32].

However, this pilot trial may face two main challenges. First, there is potential for high attrition as reported in previous online mental health intervention trials [15, 67, 68]. To anticipate this issue, all possible recruitment channels have been identified and attrition rate has been included in sample size calculations. Second, it is not possible to blind the participants in this study. Nevertheless, participants will be informed about the importance of two-arm study design and will be encouraged not to tell their peers about what group of treatment they are allocated.

## Conclusion

In summary, this study will provide important new knowledge about the preliminary effectiveness of SbS-F, a mobile application, as a digital mental health intervention and its scalability. If SbS-F shows a significant positive effect toward primary (depression) and secondary (anxiety and wellbeing) outcomes among OFWs in Macao, it has strong potential to be used by OFWs in other countries that may also experience depression and difficulty accessing mental health services. Simultaneously, this pilot hybrid type 1 study will also evaluate the possibility of SbS-F implementation and scale-up in real-world setting through collaborative work with a local community partner.

### Supplementary information

**Additional file 1.**

## Data Availability

The data that support the findings of this study are available from the corresponding author upon reasonable request.

## Appendix 1

**Table 4:** Study timeline

Step	Month											
	1	2	3	4	5	6	7	8	9	10	11	12
Preparatory works for SbS-F mobile application	**x**	**x**	**x**	**x**								
Recruiting and training e-helpers			**x**	**x**	**x**	**x**						
Internal SbS-F testing and e-helpers simulation					**x**	**x**						
Participants recruitment, screening, and randomization							**x**	**x**	**x**			
Intervention								**x**	**x**	**x**		
Follow-up and implementation evaluation									**x**	**x**		
Quantitative data cleaning and analysis										**x**	**x**	
Qualitative data analysis										**x**	**x**	
Data integration											**x**	**x**
Dissemination							**x**	**x**	**x**	**x**	**x**	**x**

## Appendix 2

**Table 5:** Overview of intervention

Content	Content overview
	**(Session 1)**
Story 1	The session starts with **information on the program** and how to use it.
Story 2	There is a **psychoeducation element** to Session 1, that is explaining that experiencing adversity can lead to low mood or anxiety and physical symptoms.
Story 3	The next part of session one, and in the interactive part, is about the **theory of change**, so how positive actions help us to feel better. We briefly mention that actions, mood and body are linked, and we mention exercise particularly triggering a naturally occurring reaction in our brains.
Interactive 1	After the recap of the session, there is **the exercise** to try doing something to make oneself feel better.
	**(Session 2)**
Story 1	**Recap** the character says that doing a small activity helped a little, but it did not last. Some users may relate to the small activity not being very helpful.
Story 2	**Psychoeducation - cycle of sadness**. This may be hard for some people to understand, so be prepared for either questions on this part, or for the user to not acknowledge it.
Story 3	Also, in this session, the younger character wants to do something more complex and so the older and younger characters **break a larger activity into steps**.
Interactive 1	In session two interactive activity part, first the older OFW character **asks if the user did the activities**.
Interactive 2	Next the older OFW character suggests that the user **do something more complex**, requiring steps.
Interactive 3	Finally, the older OFW character suggests **making a reminder** to do their activity or steps of an activity.
	**(Session 3)**
Story 1	After the recap of the previous session content, we hear of how the **younger character got nervous** or anxious about doing some activities.
Story 2	There is some **psychoeducation about anxiety symptoms** and about how slow breathing can help.
Story 3	Next, there is some **psychoeducation** and an example of a **cognitive element of low mood** (self-criticizing) and that self-affirmative statements can help.
Story 4	The character reflects on how they **wanted to give up**.
Interactive 1	**Relaxation activities**.
Interactive 2	Next the user is asked to **write down some positive self-talk** by imagining what they may say to a friend and a **confidence booster**.
Interactive 3	The user is again asked to **think of a complex activity and break it into steps**.
Interactive 4	Then, the user is asked to **write some things that may prevent them** for doing the activity then the solutions for that.
	**(Session 4)**
Story 1	After the recap of the previous session, there is some **psychoeducation on the impact of depressive symptoms on social relationships** and how that impacts the tired cycle.
Story 2	The younger character uses the techniques they learned in Session 2 (breaking activities into steps) and 3 (relaxation and positive self-talk) to help with their social activities.
Interactive 1	First the older OFW character **asks if the user did the activities**.
Interactive 2	After the recap of the session, the older OFW character asks the user to **make a list of social activities** and **break it into steps**.
Interactive 3	Similarly, to before, the older OFW character asks the user to identify any problems and solutions they may come up against.
	**(Session 5)**
Story 1	Throughout the session, the SbS **skills and knowledge are reiterated** and used to help the user recognize and prevent relapse. There is lots of **praise for completing** the intervention and some recognition that mood goes up and down, so to keep up the practice, even if we do not always feel good.
Story 2	In the session, after the recap, the younger character encourages the user to **reflect on the positive** change they have experienced.
Story 3	The character tells of how their mood did go up and down but that s/he kept up the practice, even if s/he did not always feel good.
Interactive 1	The first part of the interactive session is to **identify personal signs** that mood is worsening.
Interactive 2	The last part of the session reminds users that all their inputs in the “my activities” section can be saved for the user to remind themselves of their activities and what they have learned.

## Appendix 3

### E-helpers training syllabus

**Part 1**

1.1 Overseas Filipino Workers

1.2 Step-by-Step as e-mental health scalable intervention

1.2.1 How SbS is used and who is involved in it?

1.2.2 Behavioral activation

1.3 Step-by-Step Filipino version

1.3.1 Cultural adaption

1.3.2 Randomized controlled trials

1.3.3 Partners and its roles

**Part 2**

2.1 Being e-helpers

2.1.1 General principles of being an e-helper

2.1.2 Roles and responsibilities of e-helpers

2.2 Basic e-helping attitudes and behaviors

2.2.1 Dos and don’ts

2.2.2 Maintaining confidentiality

2.2.3 Acknowledgement of user’s emotions

2.2.4 (Not) Giving advice

2.2.5 Supporting users to identify support and coping strategies

2.3 Basic e-helping scope and support

2.3.1 Welcome contact

2.3.2 Monitoring and on-demand support contacts

2.3.3 Phone security

2.3.4 Chat or phone support contacts

2.3.5 Handling challenging contact sessions

2.3.6 How to respond to distress during a support session

2.3.7 Managing and responding to high-risk situations

2.3.8 List of services to help with other problems

2.4 Self-care, supervision, and coordination

2.4.1 Self-care and supervision

2.4.2 Supervisory meeting

2.4.3 Coordination

**Part 3**

3.1 Overview of SbS sessions, contents, and support templates

3.2 Welcome, end, and support templates

3.2.1 Onboarding, sign up, baseline assessment

3.2.2 Welcome message

3.2.3 End of program

3.2.4 Support messages (on demand only)

3.2.5 Challenging contact sessions

3.2.6 Difficult and high-risk situations

3.3 Case note templates

3.3.1 Welcome call / contact

3.3.2 Unsuccessful contact

3.3.3 Successful contact

**Part 4**

4.1 Preparation and knowing the app

4.1.1 User interface

4.1.2 E-helper e-mails

4.1.3 E-helper interface

4.1.4 Website areas for e-helper

4.1.5 Assignment of cases and updating your contact calendar

4.1.6 Prioritizing contacts

4.2 Make the contact with the user

4.2.1 Before each user contact

4.2.2 After each user contact

4.3 Basic technical skills and support

4.3.1 Technical support before account creation

4.3.2 Providing a username and password

4.3.3 Tech support after account creation

4.4 Users ending SbS and post-assessment

4.4.1 User states their desire to leave the program

4.4.2 User is inactive

4.4.3 User stops due to heightened distress

4.4.4 Post-assessments

**Part 5**

5.1 Training assessment

5.2 E-helper competency assessment

## Appendix 4

### Interview and focus group guidelines (draft)

User experiences with the intervention, including but not limited to:

1. Elements that were liked or not liked
2. Any issues around treatment acceptability
3. Possible enhancements to consider for further adaptation
4. Reason for withdrawing
5. Barriers and enablers of SbS-F adoption into community partner’s program and recommendations for addressing barriers and maximizing adoption
6. Community partner’s knowledge and beliefs about the SbS-F program
7. Participants’ perceived benefit from the intervention
8. Barriers and enablers in implementing the program and modifications needed to maximize the implementation (from participants’ and staff’s perspective)
9. Implementation of SbS-F contents in participant’s life
10. Resources, strategy and policy needed to integrate and maximize the SbS-F intervention into community partner’s program after the study ended

## Abbreviations

CONSORT: Consolidated Standards of Reporting Trials
ECAU: Enhanced care as usual
GAD-7: Generalized Anxiety Disorder-7
OFW: Overseas Filipino workers
PHQ-9: Patient Health Questionnaire
PTSD: Posttraumatic stress disorder
RE-AIM: Reach, Efficacy/Effectiveness, Adoption, Implementation, and Maintenance framework
SAR: Special Administrative Region
SbS-F: Step-by-Step program (Filipino version)
WHO: World Health Organization
WHO-5: WHO-5 Wellbeing Index
